# Bovine reproductive tract and microbiome dynamics: current knowledge, challenges, and its potential to enhance fertility in dairy cows

**DOI:** 10.3389/frmbi.2024.1473076

**Published:** 2024-10-09

**Authors:** Deepshikha Gupta, Antisa Sarkar, Yash Pal, Vishal Suthar, Aakash Chawade, Sandeep Kumar Kushwaha

**Affiliations:** ^1^ National Institute of Animal Biotechnology, Hyderabad, India; ^2^ DBT- Regional Centre for Biotechnology (RCB), Faridabad, Haryana, India; ^3^ Gujarat Biotechnology University, Gandhinagar, India; ^4^ Department of Plant Breeding, Swedish University of Agricultural Sciences, Alnarp, Sweden

**Keywords:** bovine infertility, repeat breeder, reproductive tract, microbial dysbiosis, microbiome manipulation strategies, fecundity, estrus cycle

## Abstract

The cattle production system focuses on maintaining an animal-based food supply with a lower number of cattle. However, the fecundity of dairy cows has declined worldwide. The reproductive tract microbiome is one of the important factors which can influence bovine fecundity. Therefore, reproductive tract microbiomes have been explored during the estrus cycle, artificial insemination, gestation, and postpartum to establish a link between the micro-communities and reproductive performance. These investigations suggested that microbial dysbiosis in the reproductive tract may be associated with declined fertility. However, there is a scarcity of comprehensive investigations to understand microbial diversity, abundance, shift, and host-microbiome interplay for bovine infertility cases such as repeat breeding syndrome (RBS). This review summarizes the occurrence and persistence of microbial taxa to gain a better understanding of reproductive performance and its implications. Further, we also discuss the possibilities of microbiome manipulation strategies to enhance bovine fecundity.

## 1 Introduction

The fecundity of lactating dairy cattle has been gradually declining worldwide (Sartori et al., 2002; Lucy, 2007; Lonergan et al., 2016; Funeshima et al., 2021). A plethora of factors have been reported that can influence the fecundity of dairy cattle like environmental cues, nutritional deficiency, hormonal imbalance, anatomical defects and improper management (Wiltbank et al., 1962; Reid et al., 1964; Bond and Wiltbank, 1970; Lee, 1993; Molefe and Mwanza, 2020; Holečková et al., 2021). However, in some cases, the associated factor for infertility cannot be ascertained, such RBS where an animal fails to conceive even after more than three artificial inseminations (AI) or natural breeding (Gustafsson and Emanuelson, 2002). The cause of RBS remains unexplained as these animals are clinically non-obstructive, cycling without any reproductive disorder. Diskin and Kenny (2016) defined optimal reproductive and growth efficiency as a cow producing a calf annually with an average gain of two ponds per day under normal production practices. However, these goals are difficult to meet due to reproductive losses and delays, including RBS. RBS is an expensive reproductive disorder due to the increased number of required inseminations and associated costs for non-milking open days (Funeshima et al., 2021). The issue of repeat breeder cattle remains challenging due to the inadequate understanding of disease and informative biomarkers.

Several risk factors such as management, hormonal imbalance, undetected oviductal or uterine abnormalities, and poor oocyte or embryo quality have all been linked to RBS. Some of them such as herd management, artificial insemination (AI) timing, negative energy balance (NEB), and hormonal imbalance could be mitigated to reduce RBS infertility (Pérez-Marín and Quintela, 2023). However, the occurrence and recurrence of RBS in cattle are not well understood, despite the use of normal management methods and therapies (like antibiotics, hormones, mineral supplementation, and uterine antiseptics) (Pérez-Marín and Quintela, 2023).

Recent advancements in microbiology techniques have facilitated investigators to explore the influence of different factors on the bovine microbiome such as hormonal imbalance, pathogenic invasion, environmental stress, and antibiotics usage, which can adversely affect the microbiota of the reproductive tract and may lead to microbial dysbiosis (Hashem and Gonzalez-Bulnes, 2022). A dysbiosis in the reproductive tract’s microbiome (a shift in the richness or evenness of commensal microbial populations) has been linked to numerous reproductive diseases and infertility in humans and other species (Ong et al., 2021). The present cattle research is also engaged in exploring the role of the microbiome in enhancing fertility and calving rates and developing strategies to optimize animal production practices. However, such comprehensive research is rare for the bovine reproductive tract and only a few RBS microbiome investigations have been performed. In addition, the microbiota associated with the RBS is not well known to date. This strengthens our interest in understanding if the microbiota of the reproductive tract and their shift can be used as a biomarker to better understand the relationship between the bovine microbiome and cattle reproductive efficiency, particularly RBS. Therefore, this review is focused on summaries the female reproductive tract microbiome within the vagina, uterus, placental and colostrum to understand the shift of microbiome abundance and composition in underperformed studies and discusses the emerging field of microbiome manipulation strategies for reproductive success in bovine.

### 1.1 Study design

**Figure 1:**
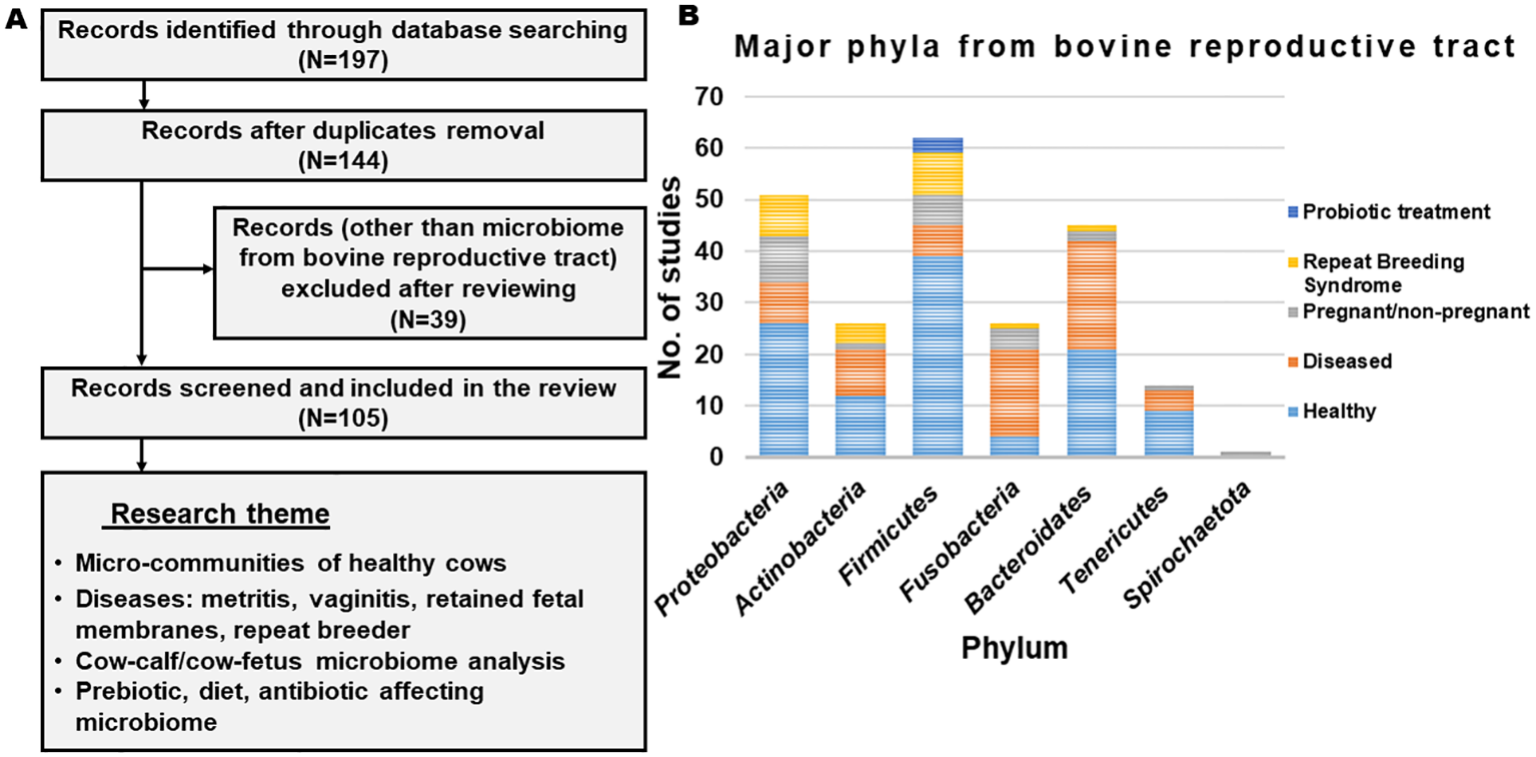
**(A)** Flowchart representing the outline of identifying, excluding and selecting the articles. **(B)** List of phyla observed across different studies.

**Figure 2:**
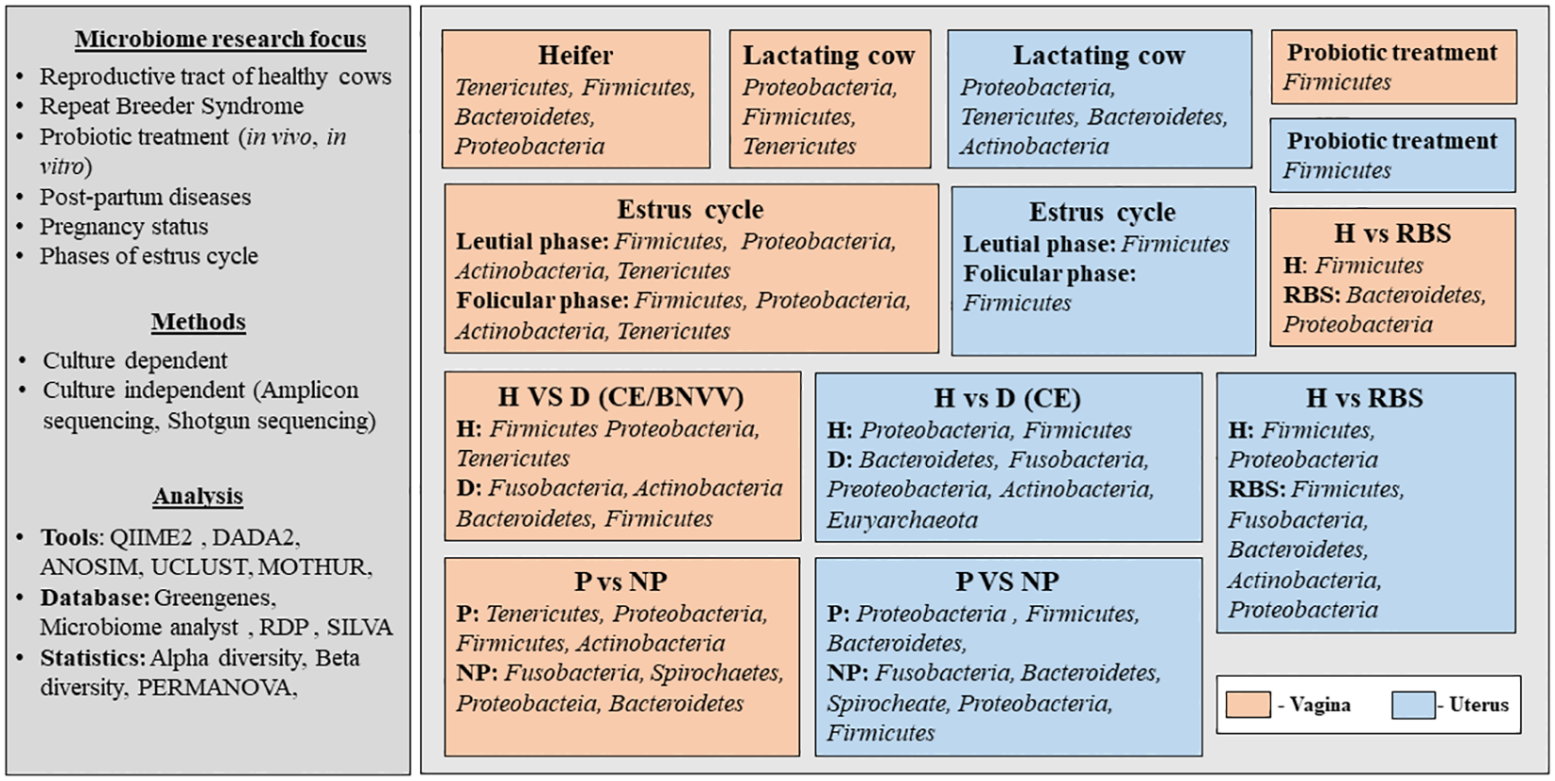
Overview of the published articles related to the microbiome abundance in the reproductive tract of cows. H, healthy; D, Diseased; P, Pregnant; NP, Non-pregnant; RBS, Repeat Breeding Syndrome; CE, Clinical endometritis; BNVV, Bovine necrotic vulvo-vaginitis.

The published studies that have discussed the microbiome from the reproductive tract of cows were screened (from 2000 to 2024) from authentic literature sources, including Pub-Med, Crossref, Scopus and Google Scholar (last accessed on 12 February 2024). The keywords used for the literature search were “microbiome and cow and reproductive tract”. Records written in English in the form of research articles or reviews were included. Initially, a total of 197 articles were obtained from the database search (**Figure 1**). After removing the duplicates and excluding the studies other than the microbiome from the bovine reproductive tract, relevant information was extracted. The selected studies were categorized based on their scientific themes as ‘micro-communities of healthy cows’, ‘microbial communities in diseased condition’, ‘cow-calf/cow-fetus microbiome analysis’ and ‘diet/prebiotic/antibiotic affecting reproductive tract microbiome’ and are summarized in **Figure 1**. **Figure 2** provides the overview of the research focus, methods and analysis tools used in the published articles to study the microbiome abundance in the reproductive tract of cows.

## 2 Microbiome of the reproductive tract of dairy cattle

The microbiome plays a crucial role in maintaining a cow’s physiological and reproductive performance. Regardless of the reproductive stage, *Ascomycota*, *Firmicutes*, and *Proteobacteria* are the abundant phyla in the bovine (Crossbred and Droughtmaster and *Bos indicus*) reproductive tract metagenome (Ong et al., 2022). However, the microbiome composition in different parts of the bovine reproductive tract has been studied in relation to various diseases such as mastitis, uterine diseases, and metabolic disorders (Khalil et al., 2022). A link between microbial dysbiosis and bovine reproductive health has been proposed which emphasizes exploring the functional aspects of the bovine reproductive tract microbiome (Ong et al., 2021). Microbiome dysbiosis can lead to an unfavorable niche for fertilization or embryo development, creating conditions to cause diseases such as subclinical endometritis, metritis, and other reproductive disorders, ultimately affecting fertility. It is speculated that microbial dysbiosis, along with infection in the bovine reproductive tract might be associated with inducing reproductive loss (Yagisawa et al., 2023). Therefore, understanding microbial taxonomy and their function in the female reproductive tract is crucial for harnessing its potential to enhance cattle fertility (Luecke et al., 2022).

### 2.1 Vaginal microbiome

The bovine reproductive tract is highly dynamic, and physiological variability may influence the existing microbial communities. Although the vaginal microbiota has been associated with fertility and reproduction, but there is a lack of evidence about the vaginal microbiome’s role in the bovine reproductive tract and pheromone production (Srinivasan et al., 2021). This is supported by case studies in humans as well (Tomaiuolo et al., 2020). However, there aren’t many reports on microbiome cattle infertility available in the public domain (Appiah et al., 2020). The bovine vaginal milieu has a nearly neutral pH (~7.3). It is well suited for the colonization of phyla *Bacteroidetes* (new name *Bacteroidota*) (Bowman et al., 2023), *Fusobacteria*, and *Proteobacteria* (Messman and Lemley, 2023). Synchronization 21 days before timed AI, showed a decrease in the number of uterine bacterial species over time in pregnant and non-pregnant beef cows (Ault et al., 2019b). Ault (2019) reported *Fusobacteria* as the dominant genera apart from lower abundance of *Acidobacteria*, *Tenericutes*, and *Verrucomicrobiota* phyla in the nonpregnant state of Angus beef cows. However, phylum *Proteobacteria* dominates, with lower abundances of *Verrucomicrobiota*, *Planctomycetes*, and unassigned phyla in pregnant Angus beef vaginal microbiome (Ault et al., 2019a). The prevalence of these phyla in cows that have experienced successful pregnancies and calving, may have a positive association with reproductive health. Giannattasio-Ferraz et al. revealed the substantial similarity between the vaginal microbiota of Nellore and Gyr cattle breed, *Aeribacillus*, *Alistipes, Bacillus*, *Bacteroides*, *Clostridium, Rikenella*, and *Ruminococcus*, being the prominent bacterial genera. *Mycosphaerella* was the dominant fungus followed by *Cladosporium* in this microbiota, while archaea were poorly represented with *Methanobrevibacter* being dominant (Giannattasio-Ferraz et al., 2019). Apart from these similar microbial profiles of cattle breeds, pathogenic fungi, especially yeast and *Penicillium*, were recently reported as colonizers in cervico–vaginal fluids of Holstein dairy cattle (Saini et al., 2019; Appiah et al., 2020). The dominant bacterial phyla in the vaginal tract of Holstein cows, are Firmicutes (37.61%), Tenericutes (29.45%), Proteobacteria (17.47%) and Bacteriodetes (13.73%), followed by Actinobacteria (0.82%) and Spirochaetae (0.45%) (Quadros et al., 2020; Brulin et al., 2023). Some species within these phyla may have antimicrobial properties that help to prevent infections, which can negatively impact fertility. These phyla might influence the immune response within the reproductive tract, helping to maintain a balanced, conducive environment for pregnancy. Sokolova (2021) reported opportunistic bacteria (*Bacillus* spp., *Enterobacter* spp.*, Enterococcus faecium, Enterococcus faecalis, Escherichia coli, Proteus vulgaris, Staphylococcus aureus, Staphylococcus lugdunensis, Streptococcus* spp.) and mold fungi (*Aspergillus* spp., *Mucor* spp., *Penicillum* spp.) from cervical swabs of Russian Black Pied cattle breed (Sokolova et al., 2021). *Ureaplasma* (the predictor genus) and *Pseudomonas* are most frequently observed in cervicovaginal mucus of nonsexually active (1.2–1.6 years old) Angus breed heifers, whereas *Alistipes*, *Bacteroides*, and *Rikenellaceae* are predictors in cows which return to estrus (De Carli et al., 2023). *Alistipes* seemed to attenuate the expression of anti-inflammatory cytokines in stressed mice, thus, it is stipulated to lower serotonin concentration thereby Knuesel and Mohajeri (2021), negatively influencing the gut-brain-reproductive axis (GBRA). The establishment of *Ureaplasma*, one of the most prevalent genera, in healthy dairy cattle’s vaginal microbiome, indicates its potential role in reproductive success (Poole et al., 2023b). Further, the presence of *Clostridium*, a common genus observed in the vagina, is presumed to be due to the close proximity of the vagina and rectum (Clemmons et al., 2017).

### 2.2 Uterine microbiome

Earlier, the uterus was considered sterile to establish or maintain pregnancy. However, recent microbiome research has challenged this theory (Molina et al., 2020). The core phyla in the uterine microbiome of heifers and cows include *Actinobacteria, Bacteroidetes*, *Firmicutes*, *Fusobacteria*, *Proteobacteria*, and *Tenericutes* (Clemmons et al., 2017; Poole et al., 2023b). They might play a role in creating a favourable uterine environment for implantation and fetal development. The most abundant bacterial families are *Lachnospiraceae*, *Porphyromonadaceae* and *Ruminococcaceae* observed in the uteri collected from Danish slaughterhouses (Karstrup et al., 2017). Some species of these families could be involved in nutrient cycling and the breakdown of organic matter, providing essential nutrients for the developing embryo. *Bacillus, Fusobacterium*, and *Porphyromonas* are the common genera, observed in both the culture-based and amplicon sequencing studies (Poole et al., 2023b). Another study found *Bacillus* and *Enterococcus* to be the most abundant genera in the uterine microbiome of Canadian Holstein Friesian cows irrespective of the estrous cycle phase, along with the lower abundance of *Lachnospiraceae, Oscillospiraceae*, and *Streptococcus* (Gobikrushanth et al., 2024). Although, *Fusobacterium necrophorum*, *Porphyromonas levii* and *Trueperella pyogenes* were located within the endometrium, on the endometrial surface and in the caruncular stroma, but were not associated with inflammation (Karstrup et al., 2017). A dysbiosis in the uterine microenvironment (higher relative abundances of *Bacteroides, Fusobacterium, Trueperella*, and *Porphyromonas*) contributes to infection (Çömlekcioğlu et al., 2024). Hummel (2022) mentioned a negative correlation between microbial abundance in the uterus and blood, which means a decreased vascular bacterial load corresponding to an increase in uterine bacterial load (Hummel et al., 2022). During postpartum, dairy cow experiences NEB since more energy is required for producing milk than acquired from feed. The uterine microbiome of cows experiencing NEB dominates with phyla *Bacteroidetes* and *Fusobacteria* as against *Proteobacteria* and *Firmicutes*, which continues to shift towards *Actinobacteria*, *Cyanobacteria*, and *Proteobacteria* later in NEB (Esposito et al., 2016). Uterine microbiota also varies between seasons; *Enterobacteriaceae, Lactobacillaceae*, *Moraxellaceae, Ruminococcaceae*, and *Staphylococcaceae* dominate during summer while, *Clostridiaceae, Bacteroidaceae, Lachnospiraceae, Moraxellaceae*, and *Ruminococcaceae* during winter (Nguyen et al., 2019). The authors articulated their prevalence at two different seasons due to their different abundances in airborne dust and bedding microbiota. During the summer, farmers use misting fans to cool the cows’ bodies; as a result, the interactions between the bedding, airborne dust and uterine microbiota may have altered over the course of the two seasons.

### 2.3 Placental microbiome

The placental microbiome exists pre-labor and is similar to the fetal oral microbiome (Poole et al., 2023b). This indicates their potential role in early colonization and immune development. Even though the placental microbiome of dairy calves has not been studied as extensively as that of humans, recent work has focused on the use of amplicon sequencing to study placental microbial transmission to the gastrointestinal tract (GIT) of neonates (Guzman et al., 2020), highlighting its potential impact on gut health immunity and overall development of the neonate. The most prevalent phylum, *Bacteroidetes* (with order: *Flavobacteriales*) and *Proteobacteria* (with order: *Rhodobacterales, Xanthomonadales, Enterobacteriales*, *Sphingomonadales, Pseudomonadales*) in the amniotic fluid is less abundant in the GIT of the calf (Guzman et al., 2020). Allantoic fluid showed a better microbial richness (Mean number of operational taxonomic unit, OTUs, 122) compared to amniotic fluid (84), intestine (63), and placenta (66) (Amat et al., 2022). However, another study observed similar bacterial richness and evenness (alpha diversity; Chao1 and Shannon index) in the amniotic fluid, inter-cotyledonary placenta, and placentomes (Moore et al., 2017). Hummel (2021) studied the influence of feed intake restriction (FIR) on the multiparous Angus cross-bred dam’s gut and placental microbiome. The study showed that FIR shaped a less robust and diverse gut microbiome, reflected in the placental microbiome as well (Hummel et al., 2021a). However, mineral supplementation improves the microbial diversity and richness of the fetal gut compared to the reproductive tract microbiome (Hummel et al., 2021b). These investigations indicate that maternal diet (e.g., FIR or mineral supplementation) can significantly impact the placental microbiome, suggesting that maternal factors play some role in shaping fetal microbial composition. Rumen microbiome shares microbial characteristics with the cotyledon, a possible microbial transmission route to the placenta as it perfuses the placenta with blood (Jeon et al., 2017). Predominating phyla in the fetal microbiome are *Acidobacteriota* (13.6%), *Bacteroidota* (5%), *Firmicutes* (16.2%), and *Proteobacteria* (55%), and the most relatively abundant genera are *Acidovorax*, *Acinetobacter*, *Brucella*, *Corynebacterium*, *Enterococcus*, *Exiguobacterium*, and *Stenotrophomonas* (Amat et al., 2022). The dominating phyla of placental tissues are *Actinobacteria*, *Bacteroidetes, Firmicutes* and *Proteobacteria* (Moore et al., 2017; Guzman et al., 2020; Zhu et al., 2021). *Moraxella, Pseudomonas* and *Ruminococcus* represent bacterial genera identified in the placenta, umbilical cord, and amniotic fluid (Zhu et al., 2021). Overall, the placental microbiome plays a crucial role in fetal development but its composition may be influenced by various factors, including maternal diet, health status, and environmental conditions. There is a need to investigate further the placental microbiome of dairy cattle and its impact on pregnancy, gestational age and establishing the offspring microbiomes.

### 2.4 Colostrum and milk microbiome

Colostrum, the first milk post-calving, imparts the passive transfer of immunity and nutrients from the dam to the calf (Lima et al., 2017). Studies have come up to determine the influence of colostrum on bacterial prevalence in the digestive tract of calves. Colostrum serves to develop an early gut microbiome before the gut facilitates its own microbial communities, as evidenced by the similarities between the colostrum microbiome and the first fecal sample (Hang et al., 2021). *Acinteobacter*, *Corynebacterium*, *Enterobacter* and *Streptococcus* are the commonly identified genera. The common phyla across colostrum samples include *Actinobacteria*, *Bacteroidetes*, *Firmicutes* and *Proteobacteria*. *Actineobacter*, *Fusobacterium*, *Bacteroides, Prevotella*, *Pseudomonas* and *Staphylococcus* are the commonly identified genera in multiparous (given birth to multiple calves earlier) and primiparous cows (giving birth for the first time). However, multiparous cows have a higher abundance of, *Actineobacter*, *Bacteroides*, *Fusobacterium* and *Staphylococcus* (Lima et al., 2017).

Milk microbiome from dairy cattle has been extensively studied since mastitis is a prevalent issue worldwide (Poole et al., 2023b). The core phyla between milk and colostrum samples include *Actinobacteria*, *Bacteroidetes*, *Firmicutes* and *Proteobacteria* suggesting that although the richness of core genera changes throughout milk production however, the overall phyla present continue to persist (Lima et al., 2017). More specifically, common genera in milk microbiome samples include *Acinetobacter*, *Corynebacterium*, *Escherichia*, *Pseudomonas*, *Lactococcus*, *Methylobacterium*, *Streptococcus*, and *Staphylococcus* (Andrews et al., 2019; Wang et al., 2020b; Gryaznova et al., 2021; Kaczorowski et al., 2022). *Escherichia*, *Staphylococcus*, and *Streptococcus* are highly abundant in infected milk samples, along with other genera varying in richness between healthy or infected samples such as *Actinobacter*, *Bacillus*, *Corynebacterium*, and *Pseudomonas* (Wang et al., 2020b; Gryaznova et al., 2021; Kaczorowski et al., 2022). Further, *Escherichia coli*, *Klebsiella*, *Mycoplasma*, *Staphylococcus aureus*, *Streptococcus agalactiae*, *Streptococcus uberis*, or *Streptococcus dysgalactiae* are observed to be associated with lower pregnancy per first AI and greater incidence of pregnancy loss (Dalanezi et al., 2020).

Despite several exciting facts and information about the contribution of microorganisms, the microbial prospect of non-obstructive cyclic infertility (RBS) in indigenous breeds has not been widely explored. Exploring the reproductive tract microbiome is critical for determining the microbiome’s functional role to bovine reproductive health. Numerous microbiome investigations have been conducted thus far, revealing tissue-specific microbiomes (vagina, uterus). However, these investigations lack an organized framework for developing a comprehensive knowledge, with the majority of studies linking a single element (diet, antibiotics, or probiotics). Dietary effects, probiotics, antibiotic effects, fertility differences, and longitudinal pregnancy studies, in combination, may have a complex influence on microbiota. Research experimental investigations based on an array of parameters may illustrate the big picture of core microbiome structure and abundance across tissues, as well as its dynamics and role in reproductive health.

## 3 Host microbiome interaction and dynamics

Microbiome investigations has revealed that the physiological process, more specifically the reproductive process, has never been the result of a single species (Howes-Mischel and Tracy, 2023). Cow as host provides diverse ecological niches for the growth of distinct micro-communities while microorganisms provide nutrients and metabolites for host growth and welfare. The micro-communities of the reproductive tract highly varies in composition, abundance and diversity depending on the reproductive health status of the cow like estrous cycle, hormonal concentration, insemination, pregnancy, calving and diseased condition (Knudsen et al., 2015a) Therefore, we hypothesize that host physiology, health status, metabolic rate, diet, and hormones influence the host-microbiome dynamics of the reproductive tract and vice-versa.

### 3.1 Microbiome dynamics during the estrous cycle, estrous synchronization and artificial insemination

The microbiome is particularly sensitive and fluctuates with cattle’s health and physiology, such as microbiome shifts reported throughout the estrous cycle of dairy cows with changes in progesterone (P4) and estradiol (E2) levels (Poole et al., 2023b). For instance, it has been observed that *Lactobacillus* is more prevalent in the follicular phase of the estrous cycle than in the luteal phase, although its relative abundance is low in dairy cattle’s vaginal microbiome (Wang et al., 2018; Quereda et al., 2020) in contrast *Streptococcus* spp. was significantly higher during the luteal phase (Wang et al., 2018). However, the guild of beef cattle’s vaginal microbiota was the same in both phases, mainly comprising *Aerococcus vaginalis, Aerococcus viridans, Escherichia coli*, *Haemophilus somnus, Streptococcus pluranimalium, Psychrobacter marincola*, and *Sphingomonas roseiflava* (Wang et al., 2018). Intravaginal P4 implants resulted in higher bacterial sequences of Family XIII AD3011 and Family XIII unclassified (Quadros et al., 2020). The P4 usage, induces hypo-estrogenism, altering the metabolic components of vaginal bacterial colonization resulting in more bacterial diversity (Wessels et al., 2019). However, vaginal bacterial community composition in Brangus heifers did not differ with E2 concentration and pregnancy status (Messman et al., 2019).

Estrus synchronization (ES) can help overcome infertility caused by prolonged anestrus. Therefore, vaginal and uterus microbiomes were also studied under ES programs. The investigations showed a lower abundance of thirteen genera (*Alistipes, Berryella, Dysosmobacter, Eggerthella, Emergencia, Ethanoligenens, Faecalibacterium, Flavonifractor, Flintibacter, Parafannyhessea, Parolsenella, Slackia, Vescimonas*) after ES (Dias et al., 2022) in beef cows. Likewise, during ES in beef cattle, different endogenous P4 and E2 concentrations are associated with uterine microbiome shift (greater Shannon’s diversity index, lower relative abundance of *Corynebacterium*, an Actinobacteria and higher that of *Ureaplasma*, a Tenericutes at high P4- low E2 as compared to low P4-high E2) and increased pH, which can impact fertility (Poole et al., 2023a). Poole (2021) correlated uterine transforming growth factor beta (TGF-β) with the relative abundance of *Treponema* and *Ureaplasma* respectively in non-pregnant and pregnant beef cows undergoing ES followed by timed AI (Poole et al., 2021). *Ureaplasma diversum*, is an obligate intracellular pathogen, capable of infecting endometrial cells and a causative organism for a spectrum of bovine reproductive failures including, endometritis, salpingitis, abortion and premature delivery, and infertility (Kim et al., 1994). Assessing intrauterine microbial communities of Holstein Frisian dairy cows soon after AI (using the Metricheck device), Ballas et al. (2021) found *Bacillus*, *Corynebacterium*, *Staphylococcus* and *Streptococcus* to be the most prevalent with respective relative abundances of 19.6%, 10.1%, 14.2%and 8.1% (Ballas et al., 2021). Researchers also noted a decreased bacterial diversity, increased vaginal pH, bacterial abundance, and vaginal inflammation after ES with the use of a controlled internal drug release device (CIDR) which was restored later (Dias et al., 2022). Another study reported members of *Aeribacillus*, *Bacteroidetes*, *Dialister, Firmicutes*, *Porphyromonas*, and *Ruminococcus* to be the dominant colonizers while evaluating vaginitis induced by progesterone-releasing intravaginal device (PRID) (Gonzalez Moreno et al., 2016).

### 3.2 The influence of host metabolism on microbiome dynamics

It is postulated that the microbiome composition undergoes transition driven by the fluxes in systemic energetics impacting the health of cows. For instance, blood energetic biomarkers from Holstein dairy cows (like albumin, beta-hydroxybutyrate, triglycerides and cholesterol) negatively correlate with taxa *Christensenellaceae* and *Pseudomonas* on the contrary, positively correlate with *Escherichia*, *Methanobrevibacter* and *Romboutsia* in the vaginal microbiome (Tardón et al., 2022). Amat (2021) claims no difference in α or β-diversity of the nasopharyngeal, ruminal and vaginal microbiota between virgin heifers raised with dams exposed to either a low (average gain of 0.28 kg/d) or moderate gain (0.79 kg/d) during the initial 84 days of gestation (Amat et al., 2021). Further, gestational age, P4 concentration in blood, and maternal nutritional plan did not alter α or β diversity of the vaginal microbiota of Brangus heifers but gestational age does change the composition of microbial taxa (Messman et al., 2021). Machado (2012) reported a higher prevalence of *Arcanobacterium* spp., *Bacteroides* spp., *Fusobacterium* spp., *Peptostreptococcus* spp., *Prevotella* spp., *Sneathia* spp. and *Ureaplasma* spp. in Holstein cows with higher uterine lavage; *Arcanobacterium* spp., *Bacteroides* spp., *Fusobacterium* spp., and *Ureaplasma* spp. in the uterus of non-pregnant cows; in contrast lower prevalence of *Anaerococcus* spp., *Parabacteroides* spp., *Peptostreptococcus* spp., and *Propionibacterium* spp. in trace mineral supplemented cows (Machado et al., 2012).

### 3.3 Microbiome dynamics under diseased conditions

Until 2010, our understanding, exclusively relying on culture-dependent studies, found a correlation between *T. pyogenes* in the uterus and clinical endometritis. After 2010, culture-independent studies explored *E. coli* to be the pioneer pathogen predisposing cows to *F. necrophorum* and *T. pyogenes* infection, which is associated with endometritis (Galvão et al., 2019a). Cows with low vaginal microbial diversity during calving are more likely to develop metritis, indicating that closely related bacteria influence the onset of reproductive infection and poor reproductive success (Bicalho et al., 2017c). Further, post-calving the vaginal and uterine microbiome coalesce for a short duration, facilitating the bacteria to travel between the two organs (Appiah et al., 2020; Poole et al., 2023b). Thus, alteration in the microbiome might influence the disease onset (Miranda-CasoLuengo et al., 2019). In line to this, Wang also reported bacilli and lactic acid bacteria (LAB) of genera *Enterococcus*, *Lactobacillus*, and *Pediococcus* from both healthy and postpartum uterine-infected Holstein dairy cows. Although, infected cows had a higher enteric bacteria population in the vagina (Wang et al., 2013).

Vaginal mucosal secretion from cows with the reproductive disorder (the clinical sign of a whitish vagina, purulent vulvar discharge (PVD), and inflamed hyperemic vulvar mucosa with granulomatous vulvovaginitis) showed the prevalence of *Alistipes*, *Bacteroides*, *Coriobacteriaceae*, *Enterobacteriaceae*, *Histophilus* and *Victivallis* as most abundant taxa (OTU). Healthy cows did not exhibit *Histophilus*, which was found in the vaginal communities of cows with reproductive disorders (Rodrigues et al., 2015). Holstein Friesian cows with a pathological puerperium, have reduced bacterial diversity with frequent prevalence of *Bacteroides* spp., *Fusobacteria* spp. and *Helcococcus* spp (Kronfeld et al., 2022). There is a report of common uterine pathogens (*Bacteroides* spp., *Escherichia coli* spp., *Fusobacterium* spp., *Prevotella* spp., and *Trueperella* spp.), potential pathogens (*Bacillus licheniformis*, *Enterococcus faecalis*, *Mannheimia* spp., *Pasteurella multocida*, *Peptostreptococcus* spp., Nonhemolytic *Streptococci* and *Streptococcus aureus*) and opportunistic contaminant (*Aspergillus* spp., *Clostridium perfringens*, *Hemolytic streptococci*, *Klebsiella pneumoniae*, *Micrococcus* spp., *Proteus* spp., *Streptococcus* spp.) (Williams et al., 2005).

Some bacteria, such as *Lactobacillus* spp., are associated with uterine health while others are associated with uterine diseases like metritis, endometritis, and pyometra, negatively impacting fertility, milk yield, and health of Holstein-Friesian dairy cows, such as *E. coli, F. necrophorum*, *Prevotella* spp., and *T. pyogenes* (Rosales and Ametaj, 2021). Holstein cows suffering from metritis showed a higher abundance of *Bacteroidetes* and *Fusobacteria* and acid stress resistance genes indicating the importance of the latter in an infected uterus for microbial survival (Bicalho et al., 2017b; Pascottini et al., 2020). In contrast, Yildirim reported *Alloprevotella*, *Campylobacter*, *Caviibacter*, *Falsiporphyromonas*, and *Veillonella* only in sick cows and a lower relative abundance of *Bacteroidota* in endometritic cows (Yildirim et al., 2022). Additionally, culture-independent denaturing gradient gel electrophoresis (DGGE) indicated the presence of *Actinobacteria* (Santos et al., 2011). Culture-dependent studies identified the presence of *Bacteroides* spp., *E. coli*, *Fusobacterium necrophorum*, and *T. pyogenes* (formerly known as *Arcanobacterium pyogenes*) in endometritic cows, while *Bacillus* spp., *Staphylococcus* spp., and *Streptococcus* spp. in the uteri of healthy cows (Galvão and Santos, 2014). Knudsen (2015) reported an association of *Fusobacteriaceae*, *Leptotrichiaceae*, *Mycoplasmataceae* and *Porphyromonadaceae* with metritis and endometritis (Knudsen et al., 2015b). Metritic cows showed increased abundance of *Bacteroides, Clostridium, Fusobacterium, Phocaeicola, Porphyromonas, Prevotella*, and *Streptococcus*; lower that of *Dietzia* and *Microbacterium*, while no change in the abundance for *Escherichia, Histophilus*, and *Trueperella* (Jeon et al., 2016; Basbas et al., 2023). Jeon et al. also pointed *Bacteroides pyogenes* as a fever-related species in metritic Holstein cows (Jeon et al., 2016). The most prevalent bacteria in uterine infection of dairy cows in southern Ethiopia are *Arcanobacterium pyogenes* (12.5%), *Escherichia coli* (45%), *Enterobacter aerogenes* (12.5%), *Fusobacterium* spp. (12.5%), *Klebsiella* spp. (22.5%), *Salmonella* spp. (5%) coagulase-negative *Staphylococci* (12.5%), coagulase-positive *Staphylococci* (30%), *Streptococcus* spp. (7.5%), *Pasteurella* spp (2.5%) and *Proteus* spp (5%) (Mekibib et al., 2024). Wagener et. al., used a Fourier-transform-infrared spectroscopy-based culturomics approach, showed that the aerobic uterine microbiota comprises bacteria belonging to 202 species, representing 76 genera, dominated by *Bacillus pumilus* (5.2%), *Escherichia coli* (11.2%), *Staphylococcus xylosus* (5.4%), *Streptococcus uberis* (4.9%) and *Trueperella pyogenes* (13.2%) (Wagener et al., 2015).

The intrauterine microbial population analysis of Holstein cows suffering from PVD showed *Bacteroidetes* to be the most abundant phyla with an increased abundance of *Fusobacteria* and a unique presence of *Trueperella* (Bicalho et al., 2017a). The vaginal microbiome of multiparous cows with PVD has a higher relative abundance of *Fusobacterium necrophorum, Porphyromonas levii, Trueperella pyogenes* with higher functional potential for protein synthesis, energy metabolism, and growth, whereas primiparous cows showed a minor difference in the microbiome. In contrast, the uterine microbiome of primiparous PVD cows has a lower relative abundance of *Bacteroides heparinolyticus* (Moore et al., 2023). Further, the relative abundance of *Caviibacter abscessus* is higher in the vagina of Holstein-Friesian cows with PVD, whereas Jersey cows with PVD have abundant *Catenibacterium mitsuokai, Finegoldia magna, Klebsiella variicola*, and *Streptococcus anginosus* (Moore et al., 2023). Studies identified indicator taxa for bovine necrotic vulvovaginitis (BNVV), such as *Bacteroidetes*, *Mycoplasma*, *Parvimonas, Porphyromonas*, and unclassified *Veillonellaceae* (Shpigel et al., 2017) and dominance by *Aggregatibacter*, *Phocoenobacter*, *Sediminicola*, *Sporobacter* and *Streptobacillus* (Swartz et al., 2014).

Moreno et al. (2022) assessed the vaginal microenvironment of healthy heifers (H) and cows with impaired reproductive performance, like metritis complex (MT), and repeat breeders (RB) in Holstein dairy cows. They revealed a shared microbiological guild but a higher relative abundance of *Bacteroidetes*, *Fusobacterium* and *Helcococcus* in MT compared with H and RB (Moreno et al., 2022). Moreno (2020) assessed the *E. coli* isolates (an important reproductive tract disease and subfertility-causing pathogen) from H, and MT or RB, assigned them to phylogenetic groups A (74%), B1 (17%) and D (9%) and observed RB strains were more represented by B1 (Moreno et al., 2020).

### 3.4 Microbiome dynamics during pregnancy/parity

A study, characterizing uterine microbiome from beef cows before AI found a lower relative abundance of phylum Firmicutes and Genus *Blautia* in the uterus of open cows. However, phylum *Tenericutes* (Genus *Ureaplasma*) increased in relative abundance of pregnant cows (Smith et al., 2023). Studies have also reported a higher abundance of *Staphylococcus aureus* and *Trueperella pyogenes* in cervical swabs from cows that have been aborted, as compared to those without any record of abortion (Anderson, 2007). Some efforts have been made to predict reproductive success and failure in beef heifers by pre-breeding microbial profiling from the vagina and faeces using the Random Forest model (Deng et al., 2019). The optimal model based on the maximum area under the curve identified predictors of pregnancy status from vaginal (*Campylobacter, Clostridiaceae*,*Histophilus somni*) and faecal samples (2 associated with Bacteroidales, 1 with *Lachnospiraceae*) (Deng et al., 2019). Not only the pregnancy state, but the parity of the cow also influences the microbiota of the reproductive tract. Ni (2023) reported a lower relative abundance of vaginal microbiota in high-parity than that in low-parity Italian Simmental cows. *Caviibacter* and *Methanobacteria* might be playing a key role in cow’s reproduction by involving in amino acid metabolism and endocrine function (Ni et al., 2023). The relative abundance of Actinobacteria is higher in primiparous Holstein cows than the multiparous cows, at the genus level, *Bacillus* and *Fusobacterium* being more abundant whereas, *Bifidobacterium*, *Lactobacillus*, *Neisseriaceae*, *Paracoccus*, *Staphylococcus*, and *Streptococcus* are less abundant in primiparous cows (Pascottini et al., 2021). Using a culture-based approach, 11.22% of bacteria showed morphophysiological characteristics of *Escherichia coli*, 88.78% *Staphylococcus* spp., and 120 isolated yeast colonies were identified to be *Candida tropicalis* (69%), *C. albicans* (24%), and *C. krusei* in the vaginal microbiota of multiparous and nulliparous Mongrel cows (Silva et al., 2019).

### 3.5 Calf microbiome derived from maternal sources

The genital microbiome is established very early in life, even before calving (Adnane and Chapwanya, 2022) and the colonization of the pioneer fetal microbiota is influenced by the maternal nutritional regime during gestation (Amat et al., 2022). Studies suggest that 46% of neonatal calf’s luminal microbiota and 41% of mucosal resembled in at-least one of the dam’s sources (oral, colostrum, udder skin, and vaginal scrapings), the majority being shared with udder skin (Alipour et al., 2018; Yeoman et al., 2018). The upper respiratory tract microbiota of calf, regardless of its age, is highly similar to the dam’s vaginal microbiota, *Bacteroides, Mannheimia, Moraxella, Pseudomonas* and *Streptococcus* being more commonly observed (Lima et al., 2019). Klein-Jöbstl et al. (2019) also found a similarity between the dam’s vaginal and calf faecal microbiome and suggested that the calf faecal microbiota is partially inoculated from the birth canal (Klein-Jöbstl et al., 2019). This points out that calf faecal microbiome inoculation might be derived from different maternal sources and the dam’s microbiome can be used for predicting calf microbiome development (Owens et al., 2021). Interestingly, the microbial influx into cow’s reproductive system occurs during mating or calving resulting in alteration of commensal microbial composition and affecting the overall fertility of cows (Adnane and Chapwanya, 2022) such as, lower abundance of LAB and high titer of opportunistic bacteria may renders the cow’s fertility (Metleva et al., 2022).

### 3.6 Other known microbiome dynamics

Immunizing pregnant Holstein heifers with formulations, containing proteins (FimH, leukotoxin, and pyolysin), inactivated whole cells (*Escherichia coli*, *Fusobacterium necrophorum*, and *Trueperella pyogenes*), or both, reduced the incidence of puerperal metritis decreasing total vaginal bacterial load (Meira et al., 2020). Treating metritic Holstein cows with ceftiofur or ampicillin decreases the uterine microbial richness, increasing evenness to become more homogeneous over time. More specifically, *Bacteroidetes* significantly increased in ceftiofur-treatment but not after ampicillin treatment however, the relative abundance of *Porphyromonas* increased with ceftiofur but decreased with ampicillin treatment (Jeon et al., 2018). *Tenericutes*, comprising *Ureaplasma* and *Mycoplasma* spp. are generally considered commensals within the lower reproductive and urogenital tract, but their occurrence within the uterus can lead to adverse reproductive outcomes (Ong et al., 2021). A study provides evidence that *Tenericutes* (including *Ureaplasma* and *Mycoplasma*) have the potential to affect fertility via both virulence and alterations of hormone concentrations within the uterine environment (Santos Junior et al., 2021). *U. diversum* alters prostaglandin production by increasing prostaglandin F2α (PGF2α) production and decreasing PGE2 (prostaglandin E2) thereby, affecting fecundity (Kim et al., 1994).

Various bovine-microbiome research investigations have shown that bovine hormone regulation impacts microbiome dynamics:

1. Hormonal changes during estrous cycle, pregnancy or lactation alter the reproductive tract microbiome (*Lactobacillus* is more prevalent during follicular phase but *Streptococcus* spp. during the luteal phase).
2. Developmental stage impacts microbiome maturation: Microbiome development and maturation are shaped by host developmental stage (e.g., neonate’s luminal or mucosal microbiota resemble dam’s colostrum, oral or udder skin microbiota, adult calf’s respiratory tract microbiota resembles dam’s vaginal microbiota).

## 4 Potential routes for microbial transmission

**Figure 3:**
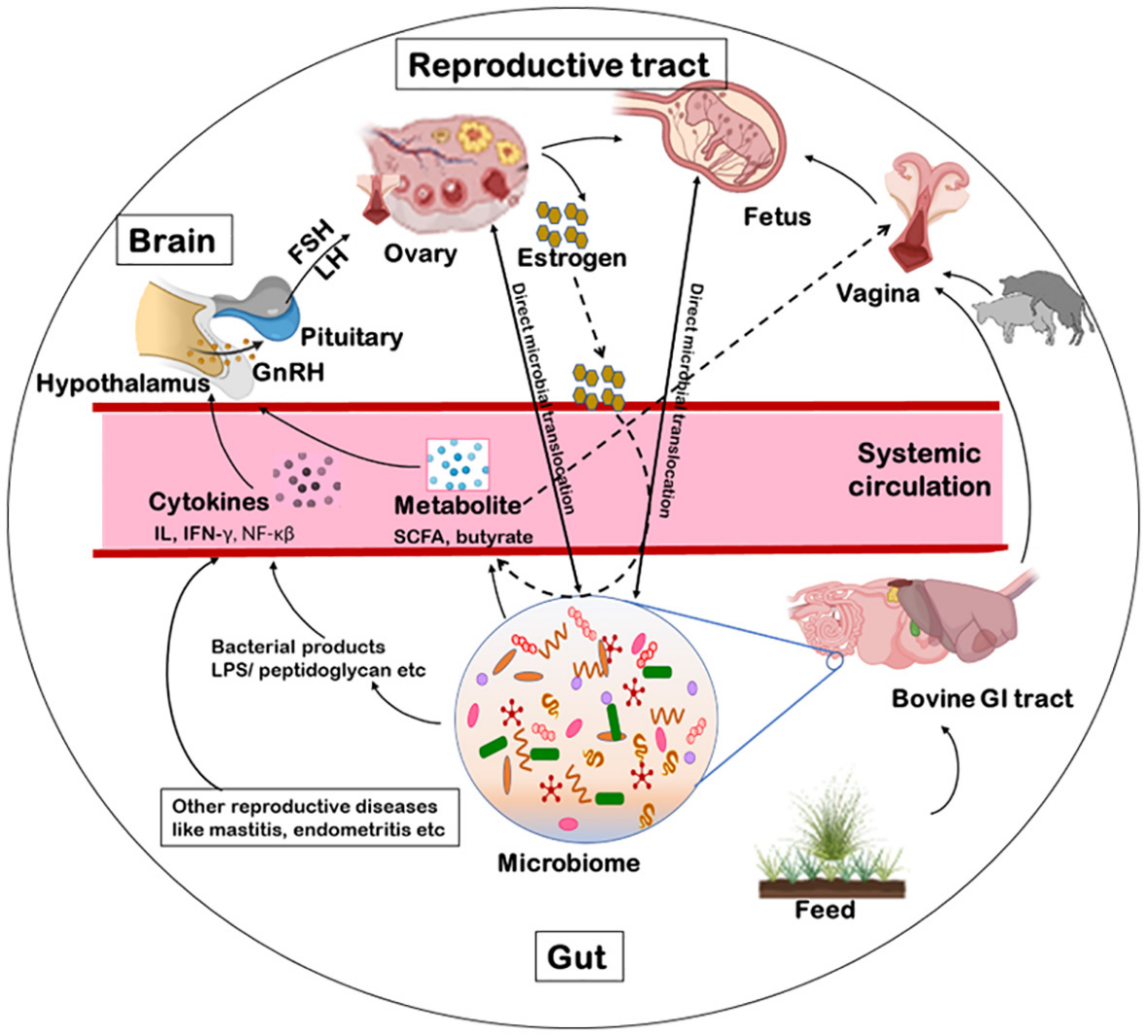
Schematics of microbial transmission routes. The feed manipulates the microbiota of the gastrointestinal tract. The microbiome excretes metabolites like SCFA, butyrate or acetate. These metabolites, via blood circulation, can influence directly the hypothalamus to release the GnRH or indirectly alter the vaginal microbiome. Some bacterial products (like LPS, peptidoglycan, exotoxin or endotoxin) or other reproductive diseases can indirectly influence the release of GnRH via immunological response. The GnRH influence the release of FSH/LH thereby, influencing the release of estrogen from the ovary and the reproduction. Another possible route for bacterial transmission is from faeces to the vaginal tract from where they can ascend to the uterus as well. Additionally, some microbial species can be sexually transmitted between animals via secretions, semen, seminal plasma, and preputial and vaginal mucus. Further, bacteria can transcend to the brain, ovary, uterus or vagina via a hematogenous route as well. GnRH, gonadotropin-releasing hormone; FSH, follicle stimulating hormone; LH, luteinizing hormone; LPS, lipopolysaccharide; SCFA, short chain fatty acids.

Female reproductive systems of cattle contain several microorganisms dwellers from an early age, even before birth (Adnane and Chapwanya, 2022) via different microbial transmission routes. Firstly, the vulva lies directly ventral to the anus thus, microbial contamination within the vaginal tract with feces is quite possible (Messman and Lemley, 2023; Poole et al., 2023b). It was argued that the location of the uterus close to the vagina, which is consistently a colonization site, would inevitably make some bacterial movement to the uterus (Baker et al., 2018). On the contrary, uterine environment may also promote microbial seeding and diseases such as endometritis. Certain bacterial species, such as *Fusobacterium*, responsible for uterine metritis, have shown a tendency to colonize in uterus of cows (Santos and Bicalho, 2012). Lietaer et al. (2021) observed a higher relative abundance of *Proteobacteria*, and a lower that of *Firmicutes* in the uterine microbiome than vaginal with the limited number of shared OTUs suggesting the possibility of bacterial transmission routes other than the transcervical (Lietaer et al., 2021). Secondly, blood seeps in the uterus after calving which may be a possible seeding source for uterine pathogens, in addition to ascending vaginal contamination *to* and *fro* (Jeon et al., 2017). Leukocytes enormously migrate to the uterus with the impending parturition and into it after parturition (Hansen, 2013). Thus, bacteria, free-floating or engulfed by monocytes/macrophages, get a fair chance for transiting to the uterine lumen, post-calving (Jeon et al., 2017). Thirdly, *Bacteroidetes* and *Fusobacteria* are the dominant reproductive tract microbiota of cows that eventually develop reproductive disease; these phyla synergistically cause reproductive disease via growth factor expression (such as leukotoxin, endotoxin, haemolysin, haemagglutinin and adhesin) and virulence (Tan et al., 1996). Transmission of these bacteria has been shown through hematogenous spread (through the bloodstream) from the gut to the uterus (Jeon et al., 2017). This can occur via either oral (Fardini et al., 2010) or the gut route allowing bacteria from mucosal sites like the oral cavity or the GIT respectively to colonize distal mucosal sites during epithelial barrier breach (e.g., gingivitis and leaky gut) (Jeon et al., 2017; Lindheim et al., 2017); and fourth is sexual transmission of pathogens via secretions, semen, seminal plasma, and preputial and vaginal mucus (Miller et al., 1994). Thus, bacterial transfer is of concern when using assisted reproductive technology (ART). A study hypothesized that *Tenericutes* ascend from the vaginal tract to the uterus during artificial insemination (AI), estrus or post-calving when the cervix is dilated (Santos Junior et al., 2021; Poole et al., 2023b). There are a few mechanisms of potential microbial transmission routes that can directly or indirectly influence the fertility of the host such as via GBRA, hematogenous spread immune response or sexual transmission as discussed further (**Figure 3**). We propose that the microbial transmission routes have the potential to influence microbiome and thus fecundity via different host gut-microbiome-metabolic axes.

### 4.1 Gut brain axis and fecundity

Harnessing the potential of microbiome to enhance cattle fecundity is an area of ongoing research. In addition, researchers are examining the role of gut bacteria in regulating the health and reproductive capabilities of cattle. However, challenges associated with studying the bovine microbiome and implementing microbiome-based interventions need to be addressed (Luecke et al., 2022). The vaginal microbiome has emerged as a crucial factor in ART and can influence the outcomes, such as recurrent implantation failure and frequent miscarriages (Günther et al., 2022). The gut microbiome, considered an extended endocrine organ, acts as an essential regulator of female reproductive health and associated diseases (Chadchan et al., 2023). Compelling data supports the relationship between gut microbial dysbiosis and ovarian dysfunction, and vice versa (Fan et al., 2020). The gut microbiome and the reproductive tract microbiome are interconnected, and their interactive effects can influence bovine reproductive health (Luecke et al., 2022). The gut microbiota has been demonstrated to significantly influence hormonal regulation by changing a series of physiological processes mediated by sex hormones (Markle et al., 2013; Baker et al., 2017) which can influence fertility, decrease conception rates, and reproductive outcomes and increase embryonic loss.

Lack of vital nutrients, such as vitamins and trace elements, can result in intestinal dysfunction and enteric dysbacteriosis in cows resulting in a malnutrition state, causing weakening of the immune system, alteration in sex hormone levels and reduced reproductive potential (Wang et al., 2021). In a study, feeding hempseed cake to Angus-crossbred heifers altered the gut microbiota (enriched genera being *Eubacterium nodatum*, *Lachnospiraceae*, *Oribacterium*, *Prevotellaceae*, *Prevotellaceae*, and *Rikenellaceae*) and vaginal microbiome (less abundant *Agathobacter*, *Cellulosilyticum*, *Clostridium*, *Negativibacillus*, *Paeniclostridium*, *Romboutsia*, *Ruminococcus gauvreauii* and higher abundance of *Fusobacterium*) was hypothesized to affect fertility and pregnancy rates in cattle (Winders et al., 2023). Another study evaluating the effect of diet composition on uterine and vaginal microbiota of beef heifers observed higher relative abundance of *Caloramator, Clostridium, Faecalibacterium, Oscillospira, Pedobacter*, *Paludibacter, Porphyromonas*, *Prevotella*, *Rhodothermus*, and *Roseburia* in vagina with the concentrate inclusion whereas, *Caloramator*, *Paludibacter*, and *Thalassospira* were the affected genera in uterus (Pickett et al., 2022). The gut microbiome plays a crucial role in nutrient metabolism and absorption in bovines, which is indirectly linked to fecundity (Deng et al., 2019). The sources and quality of feed also affect the production of ovarian hormones and the internal environment such as follicles, uterus, and oviduct, which further influence reproductive performance (Leroy et al., 2008). Additionally, studies have found that uterine pathogens (such as *Bacteroides*, *Porphyromonas*, and *Fusobacterium*) can be transmitted from the gut to the uterus through the bloodstream, highlighting the potential role of the gut microbiota in reproductive health (Jeon et al., 2017).

The gut microbiota communicates bidirectionally with the body via different microbiome-gut-organ axis (MGOA) some of which are established such as the gut-brain axis (GBA) and some are proposed like the microbiome-gut-reproductive axis (MGRA). The exometabolome of gut microbiota sends signals throughout the body to different organs, which, in turn, affect the immune system and host physiology (Welch et al., 2022). The ratio of circulating follicle-stimulating hormone (FSH)/luteinizing hormone (LH) has been connected with systemic lipopolysaccharide (LPS) and the bacterial genera *Actinobacteria*, *Bacteroides*, and *Streptococcus* (Wang et al., 2020a). A study showed that supplementing feed with short-chain fatty acids, including acetate, propionate, and butyrate, increased the secretion of LH and FSH in female Wistar rats (Olaniyi et al., 2022) and Landrace×Yorkshire sows (Xu et al., 2023). Research has demonstrated that the MGRA may be a valuable tool to enhance reproductive efficacy in cattle herds by preventing reproductive diseases and increasing hormone secretion (Welch et al., 2022).

### 4.2 The interplay of microbiome and hormone/immune response

The vaginal microbiota contributes to women’s urogenital health, still, the precise mechanisms of microbiome and hormone interplay are not very clear, even in humans (Ravel et al., 2011). However, certain bacterial families (like *Lachnospiraceae* and *Rikenellaceae*) and genera (such as *Acinetobacter*, *Bacillus*, *Oscillospira*, CF231, and 5–7NS) have been identified as signature vaginal microbiota of healthy dairy cows, with the potential for being a therapeutic target (Moreno et al., 2022). Additionally, the uterine microbiome can influence reproductive success and pregnancy outcomes by supporting to maintain the appropriate pH, providing and utilizing nutrients and metabolites, and influencing the immunological responses of the reproductive tract (Ault-Seay et al., 2023). Bovine reproductive performance is also affected by infectious diseases other than those of the reproductive tract such as mastitis (an inflammatory response of the udder tissues of the mammary gland) and endometritis (an inflammatory disease that affects the endometrium). Colonization and disease depend on the balance between the classic triad of the environment, microbial virulence and host defense system (Sheldon, 2015). The infection causes a systemic immune response, resulting in the abnormal secretion of cytokines and hormones thereby affecting the functioning of the reproductive system such as the ovary, corpus luteum (CL), uterus, and embryo (Wang et al., 2021).

**Figure 4:**
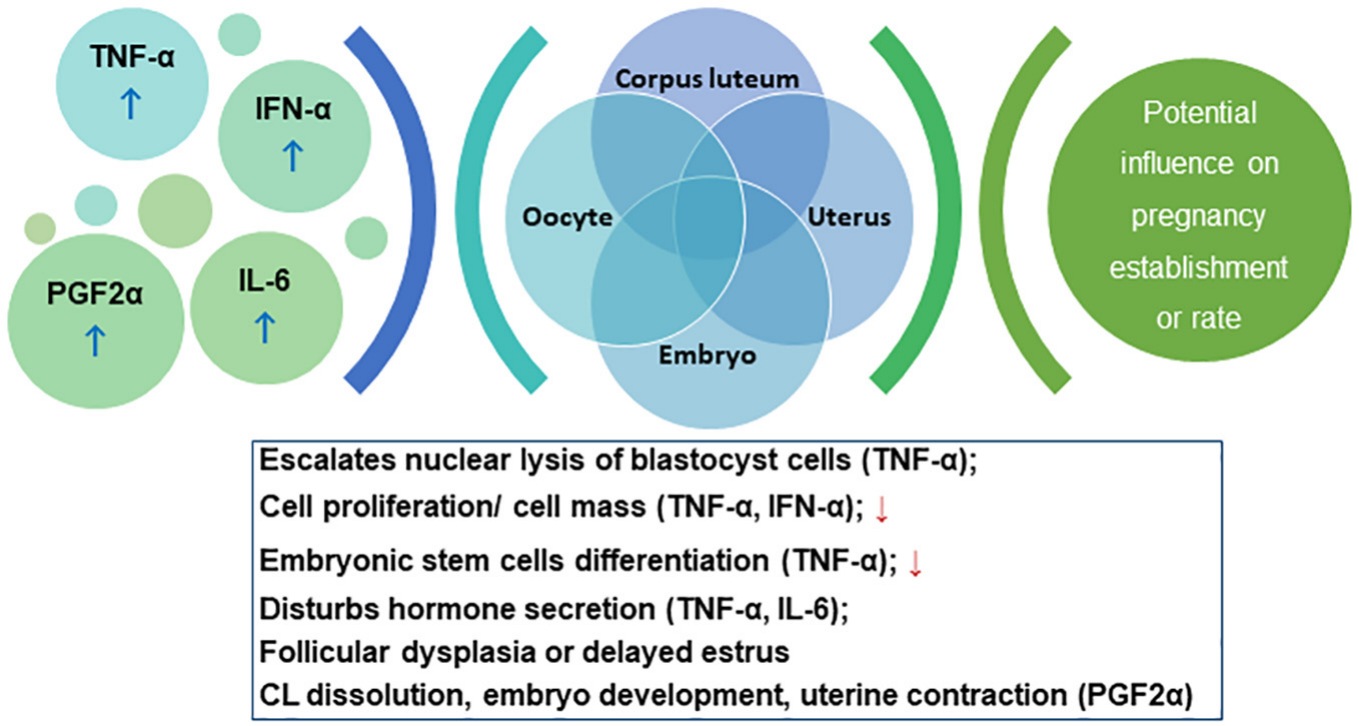
Schema for the cascade of the interplay between immunological response and pregnancy outcome. Please refer to section 5.2.

Mastitic cows show delayed estrus, reduced pregnancy rate, and higher abortion risks (Lavon et al., 2011). Etiologically, mastitis is triggered by bacteria invading the gland and producing LPS or other harmful metabolites (Wang et al., 2021), which elevates the levels of tumor necrosis factor (TNF-α), interferon-α (IFN-α), interleukin (IL-6), and PGF2α (Ślebodziński et al., 2002). TNF-α escalates the nuclear lysis of blastocyst cells, reducing cell proliferation, which decreases the inner cell masses, impairing the differentiation potential of embryonic stem cells, and decreasing the survival rate of embryos (Soto et al., 2003). TNF-α also disturbs the steroid secretion in granulosa and theca cells. Deb et al., showed that TNF-α influences oocyte function by influencing aromatase activity and E2 secretion in granulosa cells (Deb et al., 2011). The reduced steroid production may alter the follicle fluid composition, which destroys the milieu of oocytes developing in the follicles, affecting fertilization and embryonic development. IFN-α can also inhibit the proliferation of oviductal epithelial cells (Hansen et al., 2004). IL-6 constrains the proliferation and FSH-induced E2 secretion by follicles (Alpizar and Spicer, 1994). TNF-α and IL-1β stimulate the prostaglandin secretion in the endometrium, promoting luteolysis via PGF2α, and altering the endometrium proliferation. The increased PGF2α concentration promotes CL dissolution, impairing embryo development, and inducing uterine contraction, leading to pregnancy termination (Wang et al., 2021). LPS, the major component of Gram-negative bacterial outer membrane, stimulates immune responses and cytokines release, thereby affecting the HPGA and resulting in augmented follicular atresia, delayed estrus, and pregnancy failure (Wang et al., 2021). Cytokines inhibit gonadotropin-releasing hormone (GnRH) release thereby inhibiting the surge of pituitary FSH/LH and the estrogen synthesis, causing follicular dysplasia and delayed estrus (Pate et al., 2010). Cytokines such as IFN-α inhibit LH secretion and reduce the plasma P4 concentration, compromising the pregnancy rate. The cascade of events is schematically summarized in **Figure 4**.

Pregnancy establishment in bovines requires maternal immune cell modulation (Mohapatra et al., 2023). The interaction between the commensal microbiota and the immune system is multifaceted involving various mechanisms, including the production of metabolites, interaction with immune cells, and modulation of the gut barrier (Zheng et al., 2020). These interactions can have systemic effects on immune function (Neuman et al., 2015). The microbiome educates and helps to shape the immune system, particularly during early life. Exposure to diverse microbial species helps the immune system learn to distinguish between harmless and harmful substances, promoting immune tolerance and preventing inappropriate immune responses (Aldars‐garcía et al., 2021). The microbiome helps strengthen the physical barriers and enhances the production of antimicrobial peptides and mucus by specialized intestinal epithelial cells, which play a role in defending the host against pathogens (Wiertsema et al., 2021).

The reproductive tract of dairy cows has innate and adaptive immune mechanisms to protect the fertilized ovum against any potential infection (Rosales and Ametaj, 2021). Innate immunity, which is the first line of defense, involves physical, chemical, and cellular barriers, such as mucus, antimicrobial peptides, and neutrophils. Linked reduced neutrophils and lymphocyte recruitment with uterine infections in dairy cows, post-parturition (Galvão et al., 2012). Immunological adjustments to the conceptus also make the uterus more susceptible to microbial infection for example, P4 decreases uterine capacity to eliminate bacterial infections, contributing to compromised immune function (Hansen, 2013). Adaptive immunity involves antigen-specific responses, such as T cells, B cells, and antibodies (Rosales and Ametaj, 2021). Thus, the balance between pro-inflammatory and anti-inflammatory mediators is crucial for maintaining uterine health. During parturition, by losing the luminal epithelium of the endometrium, the immune defense of the uterus weakens further (Hansen, 2013). Understanding the interplay between the microbiome and the immune system opens up potential therapeutic avenues. Modulating the microbiome through interventions such as probiotics, prebiotics, and faecal microbiota transplantation (FMT) can be explored as a strategy to influence immune function and treat immune-related reproductive disorders.

The microbiome is highly dynamic and gets influenced via several transmission routes.

1. Host-microbiome metabolic axis regulates fecundity: The host gut-microbiome-metabolic axis plays a crucial role in regulating energy or hormone balance, metabolic health and ultimately fecundity. For example, hempseed cake feed alters the vaginal microbial abundance (*Agathobacter*, *Cellulosilyticum*, *Clostridium*, *Negativibacillus*, *Paeniclostridium*, *Romboutsia*, *Ruminococcus gauvreauii* and *Fusobacterium*) influencing fertility and pregnancy rates.
2. Host immune system modulates microbiome composition: Immune responses to pathogens or allergens shape the microbiome, favoring beneficial microorganisms (such as *Acinetobacter*, *Bacillus*, *Oscillospira*).

## 5 Advanced microbiome manipulation strategies

The crosstalk between the host and the host’s microbiome may facilitate or interfere with the normal functioning of the host such as providing immunity or protection against pathogens or parasites; controlling and aiding reproductive success (Gupta and Nair, 2020). Recognizing the importance of the microbiome in bovine fecundity, researchers have also begun exploring the concept of microbiome manipulation as a potential solution for unexplained infertility. This involves altering the composition of the reproductive tract microbiome to restore its balance and enhance fertility.

Based on the microecological balance theory, here, we discuss the potential microbiome extrapolation strategies that may help to restore the fecundity of idiopathic infertility of cows including RBS. Microbiome-assisted breeding, probiotics (beneficial microorganisms) administration and prebiotics (substances that promote the growth of beneficial microorganisms) usage can be explored as tools to modulate the microbiome. Probiotics, live commensal microorganisms conferring health benefits, can be administered to restore the microbial balance (eubiosis). Prebiotics, conversely, serve as food for beneficial bacteria and can help promote their growth and activity. Dietary modifications, such as increasing fiber content and reducing starch intake, can also positively influence the bovine microbiome (Yagisawa et al., 2023). Providing a balanced diet that supports the growth of beneficial bacteria and minimizes inflammation can contribute to improved reproductive health in cows.

### 5.1 Microbiome assisted breeding

Microbiome-assisted breeding is an emerging technique to fabricate the host microorganisms to shape the composition of microbial communities (Mueller and Linksvayer, 2022). It is a concept that involves manipulating the host microbiota to study the effects of specific microbial communities on host health and disease. It aims to create animal models with controlled microbiomes that can be used to investigate the interactions between the microbiota and the host by using the selective breeding approach, practiced in plants for several decades. This encourages us to think, beyond the box, if we can further extrapolate the principle of selective breeding to communities small enough to be amenable, like microorganisms (Arias-Sánchez et al., 2019). The concept is to incubate some of similar microbial ecosystems in parallel for the desired trait, allowing the populations to grow for some time, ranking them according to trait value, and then selecting the best microbial communities for the next round of seeding and selection (Arias-Sánchez et al., 2019).

One method for breeding microbiomes is to employ gnotobiotic mouse models, which can then be generalized to larger species. Gnotobiotic mice are born and raised in a sterile environment and then colonized with specific microbial communities. These models allow researchers to study the effects of defined microbiota on host physiology, disease development and progression (Darnaud et al., 2021). In the field of animal breeding, microbiome information can be leveraged to improve genetic selection and prediction models. By incorporating information about the holobiont (the host and its microbiome), researchers can potentially accelerate animal genetic improvements. It is important to note that while microbiome breeding holds promise for advancing our understanding of host-microbiome interactions, there are still conceptual and practical challenges that need to be addressed. These include issues related to the complexity and stability of the microbiome, as well as the potential for unintended consequences when manipulating microbial communities.

### 5.2 Probiotics efficacy for fecundity

Probiotics are live microbial species known to provide health benefits and help restore the natural balance of the microbiota, rendering the animal return to its normal growth and health status from potential dysbiosis (Fuller, 1989). Studies witness that probiotics can positively influence host physiology by regulating micro-ecological imbalance, modulating immunity, and antagonizing pathogens (Zhang et al., 2022). Certain known probiotic species such as *Bifidobacterium infantis*, *Lactobacillus rhamnosus* (JB-1) and *L. farciminis* exhibit anxiolytic effects improving psychiatric-disorder-related behaviors, including anxiety, depression, acute stress disorders, and obsessive disorders (Hashem and Gonzalez-Bulnes, 2022). *Lactobacillus* are presumed to be associated with increasing oocyte quality in follicular fluid and secreting reactive oxygen species which is beneficial for placental angiogenesis (Owens et al., 2020). This is the outcome of their influence on the hypothalamic–pituitary–adrenal (HPA) axis, indirectly advancing the reproductive functions by attenuating negative effects of environmental stresses (Hashem and Gonzalez-Bulnes, 2022). Probiotics (*Bifidobacterium longum* CECT7347, *Lactobacillus gasseri* OLL2809, *Lactobacillus reuteri* ATCC PTA 6475, *Lactobacillus rhamnosus* CECT8361) have been also found to improve the immune responses and inflammatory status of the reproductive tract. The administration of probiotics (*Lactobacillus*) in a mouse model resulted in the reduction of endometritic lesions by improving endometrial epithelial cells barrier function, boosting the activity of natural killer cells and interleukin-12 levels (Molina et al., 2020). Thus it is suggested to use probiotics as a way forward to replenish the commensal bacteria and lower the risk of reinfection (Cribby et al., 2008). *Clostridium butyricum* effectively suppressed inflammatory responses of uterine tissues by significantly decreasing the microbial loads and alleviating the reproductive outcomes of *Escherichia coli* induced endometritis in mice (Mun et al., 2022). The probiotic *Lactobacillus* strains have been shown to disrupt bacterial vaginosis (BV) and yeast biofilms with preventive recurrences (Cribby et al., 2008). A recent study showed that probiotic bacteria (*Lactiplantibacillus plantarum* KUGBRC and *Pediococcus pentosaceus* GBRCKU) isolated from the reproductive sites of buffaloes and cows may have the ability to combat endometritis (Gohil et al., 2023). Another study highlighted the potential of probiotics in the management of reproductive tract microbiota in cattle (Hashem and Gonzalez-Bulnes, 2022), and suggested that certain genera in the vaginal microbiota such as the abundant *Bacteroidetes* and lower abundance of *Mycoplasma*, *Parvimonas, Porphyromonas*, and unclassified *Veillonellaceae* was associated with bovine necrotic vulvovaginitis. Earlier, another study highlighted the lower occurrence of purulent vaginal discharge by intravaginal LAB probiotics administration (Ametaj et al., 2014). Several benefits of intravaginal LAB in dairy cows have been observed, such as lowering the incidence of uterine infections, expediting uterine involution, increasing milk yield, and improving immune responses (Rosales and Ametaj, 2021). Sandra Genís reported the promising potential of pre-calving intra-vaginal administration of LAB probiotics as a preventive treatment against metritis in dairy cows (Genís et al., 2017). **Table 1** summarizes the studies investigating the influence of LAB (probiotics) on cattle’s reproductive performance. Considering the importance of probiotics, LAB biomass is produced in designed culture media to maintain their beneficial properties for treating bovine reproductive infections (Miranda and Nader-Macías, 2023). Furthermore, probiotics have been investigated for their role in reducing production losses from heat stress in cattle, which can negatively impact reproduction success. In a study, yeast probiotic supplementation was found to be effective in mitigating the adverse effects of heat stress on productivity and reproduction in beef and dairy cattle (Broadway et al., 2020). Several studies have suggested that an increase in air temperature (heat stress) can exert an immensely negative effect on the reproductive performance of livestock (Wolfenson et al., 2000; De Rensis and Scaramuzzi, 2003; Dash et al., 2016), adversely impacting oocyte growth, maturation, fertilization, embryonic development, and implantation (Takahashi, 2012). However, the exact mechanisms to mitigate the effects of heat stress using probiotics is not well understood for fertility. It is speculated that it may enhance nutrient utilization and immune function, by modulating the gut microbiota or though common LAB mechanisms of action, such as the production of organic acids, bacteriocins, and hydrogen peroxide, competition with pathogens, adhesion to mucosal layers, and immune modulation (Peter et al., 2018).

**Table 1:** Studies showing the effect of probiotics on cattle’s reproductive performance.

Host	Probiotic	Observation	Reference
Holstein dairy cows	Weekly intravaginal infusion (staring from 7d prepartum to 7d postpartum) of lyophilized LAB amalgam comprising *Lactobacillus sakei* FUA3089, *Pediococcus acidilactici* FUA3138, and FUA3140 (10^8^−10^9^ CFU/dose)	• Higher NEFA concentration in blood. • Higher IgG concentration but lower haptoglobin in milk • Higher milk production and feed efficiency of transitioning dairy cows	(Deng et al., 2016)
Holstein dairy cows	Weekly intravaginal infusion (staring from 14d prepartum to 7d postpartum) of lyophilized LAB mixture of *Lactobacillus sakei* FUA3089, *Pediococcus acidilactici* FUA3138 and FUA3140 (10^8^−10^9^ CFU/dose)	• Smaller cross-sectional area of gravid horn and uterine body 14 d postpartum. • Increased serum progesterone concentration, indicating earlier resumption of ovarian cyclicity.	(Deng et al., 2015)
Holstein Cows	A cocktail of LAB (*Pediococcus acidilactici*, *Lactobacillus rhamnosus*, and *Lactobacillus reuteri*) with a respective ratio of 25:25:2 tested *in vitro* and *ex vivo.*	• Lower infection of *E. coli* in uterus explants • Lower acute inflammation by *E. coli*	(Genís et al., 2017)
Late pregnant cows (Holstein)	Weekly (starting from 7d prepartum to 28d postpartum) intravaginal administration of lyophilized LAB admixture (*Lactobacillus sakei* FUA 3089, *Pediococcus acidilactici* FUA 3140, and *Pediococcus acidilactici* FUA 3138 (10^10^–10^12^ CFU/cow))	• Lower purulent vaginal discharge incidence, 21d postpartum • Lower plasma haptoglobin concentration, an acute phase protein often associated with uterine infections • Enhanced milk production in multiparous cows	(Ametaj et al., 2014)
Cows with subclinical endometritis (Holstein)	Intrauterine administration of LAB, *Lactobacillus buchneri* DSM 32407, 24-30 days postpartum.	• Higher first-service pregnancy rate of subclinical endometritic cows. • Lower endometrial mRNA expression levels of many pro-inflammatory factors.	(Peter et al., 2018)
Buffalo with endometritis	Intravaginal administration of *Lactiplantibacillus plantarum* KUGBRC or *Pediococcus pentosaceus* GBRCKU (40 × 10^8^ CFU/ml)	• reduced duration of healthy estrus induction	(Gohil et al., 2023)

CFU: Colony-forming units; LAB: lactic acid bacteria.

### 5.3 Prebiotics modulating the microbiota (prebiotics featured organic compounds have been tested as prebiotics)

The extensive use of antibiotics is one of the reasons to cause disturbance in natural microbiota. Therefore, various investigations have been started to manipulate environmental conditions using different combinations of diet and prebiotics, i.e., feed that can selectively promote the growth of specific and desired members of the microbiome to restore the beneficial microorganisms in the niche. Further, the role of nutrition and prebiotics was investigated thoroughly to target the composition and the metabolic activity of the microbiome to understand the impact on the host’s health. The anti-enterococcal and antioxidant activity of diethyl ether extract of *Leptolyngbya* sp. HNBGU 003 (DEEL-3) may be attributed to the phenolics, which may be isolated and developed as food additives (Tyagi et al., 2021). Intra-uterine administration of Oyster glycogen500 µg in 500ml PBS effectively cured the endometritis in repeat breeder cross-bred cows by improving the conception rate from first service (Solanki et al., 2019). Injecting pegbovigrastim (15 mg) subcutaneously 7d before calving and 24 h within calving did not alter the vaginal microbiome however, the Chao1 and Shannon diversity indices decreased in metritic cows (Galvão et al., 2019b). Rumen-protected choline fed Holstein cows (CholiGEM™ 15 g/d from 21 d prepartum to 30 g/d 21 d postpartum) reduced *Fusobacterium*, a common pathogen associated with metritis, in vaginal discharge microbiome (Marques et al., 2023). A study evaluating the effect of chitosan microparticle intrauterine infusion (three doses of 24g/40 ml, on alternate days) to Holstein cows with metritis reported the uterine microbiome progression towards a healthy state (Galvão et al., 2020).

High starch and low fiber diet have been associated with dysbiosis in the bovine gut (Thoetkiattikul et al., 2013; Yagisawa et al., 2023). This dysbiosis can lead to systemic inflammation, disrupting the reproductive processes in cows. Additionally, stress, such as heat stress or transportation, can also impact the microbiome and fertility in cattle (Chen et al., 2021). It is observed that oral administration of acetylsalicylic acid reduced the fetid vaginal discharge prevalence, 7 days postpartum (Rosales and Ametaj, 2021). Further, oral administration of anti-inflammatory drugs, granulocyte colony-stimulating factor, and interleukin-8 (IL-8) can serve as alternatives for uterine inflammatory responses (Rosales and Ametaj, 2021). It is also evident that neutrophil count reduces during uterine infection. Intrauterine infusion of recombinant bovine IL-8 (rbIL-8) is reported to enhance neutrophils proportion in the uterine and vaginal lumen thereby lowering the metritis prevalence (Bicalho et al., 2019).

Further, to deal with the situation of dysbiosis, the studies have come up with a “phytobiotic” formula that combines phytocompounds with probiotics. In a study, investigators aimed to study the effect of the plant extract (such as phenolic compounds of Echinacea, Lapacho and Llantén), probiotics (like *Lactobacillus gasseri*, *Lactobacillus johnsonii, Lactococcus lactis* and *Weisella cibaria*) and vitamins combination on LABs. They found Lapacho and Malva stimulate the growth of most LAB however, assessed concentrations did not inhibit the growth of most of the pathogens responsible for endometritis (Miranda and Nader-Macías, 2023).

### 5.4 Transfaunation, an alternative tool for microbiome manipulation?

Another avenue of research involves microbiome transplantation, where the reproductive tract is inoculated with a healthy microbiome from a fertile cow. This method aims to restore the microbial balance and improve fertility indices (Shalaan et al., 2023). Transfaunation refers to the transfer of ruminal fluid or contents from a healthy donor animal to another animal, typically to restore or improve rumen function (Welch et al., 2022). While transfaunation is primarily used to address rumen-related issues in cattle, however, no information is available about its impact on bovine reproductive efficiency in the public domain (Welch et al., 2022). A study compared the difference in feed efficiency before and after transfaunation, to examine the extent of microbial community establishment after exchanging rumen content between animals with different feed efficiency (Zhou et al., 2018), and observed that *Coriobacteriaceae*, *Coprococcus*, and *Lactobacillus* were the most responsive and tunable. In another study, transfaunation from healthy animals to the rumen of cows suffering from indigestion significantly improved the feed intake, milk yield, rumen pH, and protozoal count and activity as compared with the non-transfaunated cows (Galbat and Keshta, 2020). The available research does not provide direct evidence linking transfaunation to enhance bovine fertility. However, transfaunation may indirectly contribute to reproductive health by improving rumen function and nutrient absorption. Focused studies are needed to fully understand the various factors, their effects and transfaunation mechanisms to specific reproductive outcomes such as the selection of appropriate donor microbiota, the timing and method of transplantation etc.

### 5.5 Nutrition, rumen microbiota, and possible implications on fertility

The gut microbiome normally remains stable over time, it serves as a protective mechanism against infections and other disease-causing agents in the host (Kamada et al., 2013; Wolf et al., 2021). The intricate population of microorganisms known as the rumen microbiome, which inhabits ruminants is crucial to the effective digestion and utilization of feed, ultimately impacting feed efficiency. The study conducted by Franco-Lopez et al. (2020) highlights the significant contribution of ruminal microorganisms in the production of essential vitamins such as A, K, and B12 (Franco-Lopez et al., 2020). Furthermore, He et al. (2019) demonstrated the production of enzymes particularly cellulase (the enzyme responsible for the conversion of plant cell wall’s cellulose into simple carbohydrates) by these microorganisms, showcasing their involvement in breaking down complex polysaccharides and contribution to the overall nutrient availability for the host animal (He et al., 2019).

In cases where the rumen experiences dysbiosis, an imbalance in the microbial community, the efficient utilization of provided feed is compromised. Xue et al. (2020) emphasize that dysbiosis can lead to decreased energy retention in the rumen, prompting increased energy supply through the breakdown of glucose and fatty acids by the epithelium (Xue et al., 2020). Moreover, dysbiosis inhibits the growth of ruminal epithelial cells, potentially leading to intestinal tissue damage and enhanced permeability. Fu et al. (2022) added that dysbiosis may facilitate the migration of bacteria to other tissues or organs, elevating the risk of metabolic diseases, compromising the defensive capabilities of tissues and organs, and increasing susceptibility to infectious diseases (Fu et al., 2022). Metagenomic analysis of different studies has connected rumen microbiome with feed efficiency (Kruger Ben Shabat et al., 2016; Li and Guan, 2017; Ribeiro et al., 2017; Paz et al., 2018; Schären et al., 2018; Li et al., 2019; Wang et al., 2019; Ahmad et al., 2020). The influence of nutrition on reproductive health has also been extensively studied. Reid et al. (1964), Bond and Wiltbank (1970), and Wiltbank et al. (1962) have identified a low amount of nutrition as a factor contributing to infertility in female lab-grown animals (Wiltbank et al., 1962; Reid et al., 1964; Bond and Wiltbank, 1970). Nutritional deficiencies in protein and vitamin A (Guilbert, 1942) and phosphorous, (Tuff, 1923) have been linked to infertility in cows (Tuff, 1923; Guilbert, 1942). Bentley and Phillips (1951) and Wilson (1966) found that a lack of manganese further exacerbates reproductive challenges in animals (Bentley and Phillips, 1951; Wilson, 1966). Building on these findings, McClure (1968) directly connected nutritional insufficiency to bovine infertility, consolidating the understanding of how the nutritional status of animals can profoundly impact reproductive outcomes (McClure, 1968). This interplay between the rumen microbiome, nutrition, and reproductive health underscores the intricate relationships within the physiological processes of ruminant animals, providing valuable insights for effective livestock management and production practices. Pacheco-Torres’ (2022) study on healthy cow faeces revealed the presence of bacterial communities of veterinary importance (Pacheco-Torres et al., 2022). For instance, Campylobacter fetus is primarily found in the intestinal (faecal samples) and vaginal tracts of cattle and causes spontaneous abortion and infertility in cattle (Sahin et al., 2017). Overall, it is inferred that the host diet shapes microbiome composition: A high-fiber diet favors the growth of fiber-degrading microorganisms (*Lachnospiraceae*, *Ruminococcaceae*, and *Fibrobacteraceae*) and ultimately the microbial abundance in the reproductive organs.

## 6 Conclusion

Bovine reproductive efficiency is a major concern, particularly in the case of RBS, where the associated factors are unknown and the problem remains unmanageable due to the limited understanding of disease biology and a lack of diagnostic biomarkers. Hormonal changes, environmental stress, nutritional conditions, and diseases have been investigated to understand the dynamics of the bovine reproductive tract and linked with RBS cases. Investigating the interactions between the reproductive tract microbiome and the host immune system, reproductive hormones, and other factors can help to elucidate the mechanisms underlying reproductive health and fecundity. Some studies provided valuable insights into the placental microbiome and its potential role in fetal development however, examining the long-term consequences of alternating the placental microbiome on offspring health and disease susceptibility is still elusive. Further, there is a need to develop strategies to modulate the placental microbiome to improve fetal outcomes and reduce the risk of pregnancy complications. By addressing these research questions, we can gain a deeper understanding of the complex relationship between the reproductive tract microbiome and bovine reproductive health, paving the way for improved reproductive management practices and interventions. The impact of these studies can be bolstered by accompanying metatranscriptomics, metaproteomics and metabolomics approaches to quantify microbial genes, proteins and metabolites respectively. Investigations have shown promising results for breeding practices by administering probiotics, prebiotics, transfaunation or modulating the nutritional regime and this seems to be promising field. That being said, more *in vitro* as well as *in vivo* experiments need to be planned that can challenge microbiome research and aid in identifying more probiotic species for the reproductive success. Thus, more integrative approaches are needed to elucidate the interplay between the bovine reproductive tract microbiome of RBS cows and their physiology, endocrinology, and immunology, using advanced molecular and bioinformatic tools to decipher the taxonomic, functional, and metabolic contributions of the microbiome. Furthermore, longitudinal and comparative studies need to be carried out to monitor the microbiome dynamics and identify the key microbial signature associated with reproductive success and failure.

## 7 Future prospects of bovine microbiome investigations

Microbiome manipulation strategies, such as microbiome-assisted breeding, probiotics, prebiotics, and microbial transfaunation, can be explored as potential tools to modulate the reproductive tract microbiome and enhance bovine fertility. These approaches may help to reduce idiopathic infertility and improve animal welfare. Therefore, some of the research arena need attention

- Microbial interactions and their impact on reproductive performance at the molecular and cellular levels are not well understood. Thus, more research is required to elucidate the causal mechanisms of microbiome dysbiosis and bovine infertility to identify potential biomarkers and targets for diagnosis and interventions.
- The placental microbiome’s effect on conception to calving is still being studied in farm animals. However, understanding the composition, diversity, and function of the placental microbiome, as well as its interactions with the maternal and fetal microbiomes, is critical for the calf’s health and development. More targeted research is required to develop microbiome manipulation strategies and methodologies to optimize and evaluate the safety, efficacy, and feasibility.
- Due to the significant variability and diversity of the microbiome across different breeds, geographies, and production systems, a consortium-level effort is necessary to develop global microbiome profiles and functions against various influencing factors pinpointing the modulating factors for the type of infertility.
- Eventually, a comprehensive understanding of the bovine reproductive system may be possible through the integration of microbiome data with other omics data, such as, genomics, transcriptomics, proteomics, and metabolomics. This will also enable more precise breeding and management strategies.
- We also suggest exploring microbial-based therapies to manipulate the environment (high fiber diet or usage of prebiotics to promote the prevalence of selected microbial members); the practice of animal rotation (based on the principle of crop rotation) to suppress the growth of pathogens and minimize antibiotic use, and administer probiotics to stabilize symbiotic microbiota. This will advance our knowledge of the resilience of microorganisms, coping with their impacts.
